# Golf trainer safety practises in indoor simulators: impact on trust and exercise continuation

**DOI:** 10.3389/fpsyg.2025.1551831

**Published:** 2025-07-17

**Authors:** Junghoon Ha, Doojin Kim, Kwangwoo Nam

**Affiliations:** ^1^Department of Youth Guidance and Sport Education, Korea National Sport University, Seoul, Republic of Korea; ^2^Department of Management, Hankuk University of Foreign Studies, Seoul, Republic of Korea; ^3^Department of Physical Education, Republic of Korea Naval Academy, Changwon-si, Republic of Korea

**Keywords:** training, safety management, satisfaction, continuing exercise, instructional safety, indoor golf simulator, trust

## Abstract

**Introduction:**

This study examines the relationship between safety management, trainer trust, training satisfaction, and exercise continuity in golf lessons, focusing on Korea's growing participation in sports amid rising safety concerns.

**Methods:**

Data were collected via convenience sampling from 215 adults participating in golf lessons at an indoor driving range with a golf simulator in Seoul, Korea. After excluding 13 unreliable responses, 202 responses were analysed using IBM SPSS Statistics 21 and AMOS 21. Frequency analysis, confirmatory factor analysis, correlation analysis, and structural equation modelling were conducted.

**Results:**

Participants' perception of the trainer's safety management positively affected their trust in the trainer (*t* = 13.727, *p* < 0.001) and their training satisfaction (*t* = 14.402, *p* < 0.001). However, perceived safety management did not show a statistically significant direct effect on exercise continuation (*t* = −0.502, *p* > 0.05). Trust in the trainer was positively associated with participants' intention to continue exercise (*t* = 4.406, *p* < 0.001), whereas training satisfaction was not (*t* = 0.323, *p* > 0.05).

**Discussion:**

Emphasising safety and leadership skills among golf trainers is crucial for fostering a safe exercise environment. This can enhance trust and satisfaction among participants, thereby promoting their commitment to an exercise routine and positively impacting trainer retention. These findings highlight the need for improved safety training protocols for golf instructors in indoor facilities to enhance learner retention.

## 1 Introduction

In 2021, the number of golf participants in South Korea reached ~11.76 million, a 46% increase compared to 2017 (Korea Golf Association, 2023). This growth is largely attributed to the COVID-19 pandemic, which began in 2020. Golf was perceived as a safe sport during the pandemic due to minimal physical contact, individual practise opportunities, and an outdoor setting (Jun et al., 2023). Amid restrictions on public activities, increased media exposure further boosted golf's popularity across various age groups. Once seen primarily as a networking tool, golf has since evolved into a health-promoting exercise and leisure activity. This shift has resulted in increased participation, especially among younger generations, including the MZ generation (Ha and Kim, 2021).

The growing golf population has resulted in an increase in golf academies and indoor driving ranges, raising consumer expectations for high-quality facilities, services, and instruction (Kim, 2020). Indoor golf lessons using simulators, in particular, have gained popularity. As a result, the role of golf instructors has expanded from technical training to include the responsibility of ensuring a safe and effective learning environment. Safety management, in this context, involves instructors' proactive efforts to prevent accidents, control hazards, and manage instructional spaces while delivering safety education.

Recent safety incidents in golf settings have included drownings while retrieving balls from water hazards and serious injuries from disregarding caddies' instructions. Indoor practise facilities have also experienced accidents due to improper equipment use and poor spatial awareness (Kim, 2023). Safety in sports involves not only accident prevention but also system resilience, organisational culture, and human–technology interaction (Dekker, 2011; Leveson, 2012; Hollnagel, 2014). In golf education, instructors must identify risks and manage the learning environment. In South Korea, where most golf instruction occurs indoors in confined spaces, accidents are often caused by non-compliance with safety rules, improper equipment handling, and inadequate space management. Therefore, systematic safety education and environmental management, led by instructors and facility operators, are critical. These tasks are not only operational tasks but also fundamental to teaching competency (Ha and Kim, 2022).

Despite increasing research on coaching methods, few studies have examined safety management as a trust-building competency in golf instruction. Therefore, this study examines safety management as a key factor in golf instruction and its impact on training trust, training satisfaction, and continued exercise.

Training trust refers to a learner's belief in an instructor's competence, integrity, and intentions within a sports context. It develops through long-term interpersonal interactions and is shaped by the instructor's professionalism, communication, and leadership (Cho et al., 2005; Nam and Cho, 2014; Yoon and Nam, 2023). Without trust in the coach, instructional effectiveness declines, and skill development becomes more challenging. Previous research has identified coaching trust as a key factor in promoting motivation, engagement, and coaching satisfaction (Kim et al., 2019; Cho, 2021).

Training satisfaction is the learners' emotional and subjective assessment of their training experiences. It is influenced by leadership, coaching style, and interpersonal interactions and is strongly linked to coaching trust (Yoo, 2015; Min, 2022). High coaching satisfaction enhances instructional effectiveness and promotes continued participation (Choi and Lee, 2015; Bum and Lee, 2016; Ha and Kim, 2022; Mu and Jeong, 2024).

Exercise adherence refers to an individual's psychological and behavioural commitment to consistently engaging in physical activities (Weinberg and Gould, 2010). It encompasses both physical involvement and emotional attachment to the activity, often linked to health benefits, stress relief, and enjoyment (Dishman, 1994; Lee et al., 2018). In golf settings, adherence reflects how well instructors and facilities meet learners' expectations. As such, adherence may also serve as an outcome measure for assessing instructional competency, particularly in safety management.

While previous studies have examined how training competencies like leadership and communication impact trust, satisfaction, and adherence, few have focused on safety management as a distinct instructional competency; based on the literature review, it is likely that trainers' safety management positively affects participants' trust in the trainer, training satisfaction, and intention to continue exercise. This study empirically analyses the relationships among golf trainers' safety management, trust in the trainer, training satisfaction, and participants' intention to continue exercising. The findings highlight the importance of safety management in instructional effectiveness and offer foundational insights for promoting sustained participation in golf instruction. Understanding these relationships is important for enhancing instructional quality and fostering long-term engagement in formal sports settings.

## 2 Conceptual framework

### 2.1 Effects of safety management on trust in the trainer, satisfaction, and continuing exercise

In 2021, South Korea reported 1,468 golf course accidents, a 2.2-fold increase over the past five years (Korea Ministry of Culture, Sports and Tourism, 2023). While this rise may be linked to increased golf participation, it also highlights a gap in safety preparedness and awareness relative to the growing number of participants. As a result, golf course managers and instructors must recognise the inherent risks in both instructional activities and games and develop the necessary safety management skills to address potential injuries or accidents effectively (Ha and Kim, 2022).

Safety management involves eliminating risks related to physical activity and ensuring a safe environment (Park, 2001). In this study, safety management is divided into two dimensions: Facilities and Equipment for Safety, which includes managing training equipment, securing safe spaces, and providing safety guidelines for facilities; and Training for Safety, which involves conducting warm-up exercises, advising against risky behaviours, adjusting exercise intensity, and informing learners of emergency response procedures.

Prior research indicates that learners' perceptions of safety management in college physical education classes positively affect their physical, psychological, and social satisfaction (Lee et al., 2019). Additionally, effective safety management by physical education instructors enhances learners' willingness to continue participating in exercise (Ha and Park, 2023; Seo and Woo, 2025). While few studies have specifically explored safety management as a core coaching competency, substantial evidence suggests that instructor-related factors—such as expertise, coaching knowledge, communication style, and leadership—significantly impact coaching trust (Choi et al., 2013; Nam and Cho, 2014; Jang and Oh, 2014; Yoon and Nam, 2023). These findings imply that safety management may also play a key role in fostering participants' trust in the trainer.

The following hypotheses are proposed based on theoretical and empirical considerations:

H1: The golf trainer's safety management positively influences participants' trust in the trainer.
H2: The golf trainer's safety management positively influences participants' training satisfaction.
H3: The golf trainer's safety management positively influences participants' continuation of the exercise.

### 2.2 Effect of trust in the trainer on continuing exercise

Education relies on the interaction between trainers and participants, with trust in the trainer being crucial to this process. When participants trust their trainer, they are more likely to remain engaged, even in challenging circumstances, as trust offers emotional support and boosts motivation, leading to improved educational outcomes (Kim, 2007). In contrast, the absence of trust can cause participants to resist instruction, hindering their ability to internalise techniques or strategies and reducing training effectiveness (Nam and Cho, 2014). Therefore, participants' trust in the trainer is essential for achieving shared goals and enhancing educational effectiveness.

Previous studies have highlighted trust in the trainer as a key factor in enhancing participants' intention to continue exercising. For example, Kim and Kim (2024) found that learners' trust in golf trainers positively influenced exercise adherence. They suggested that trust reflects the participants' willingness to accept evaluation, recognise personal limitations, and commit to sustained efforts for improvement. Similarly, Kim et al. (2019) showed that athletes' trust in their judo coaches significantly impacted their motivation to persist. Athletes who trust their coaches through fair evaluation, recognition, and shared goals are more likely to overcome challenges and maintain consistent participation, even under physically and mentally demanding conditions.

The following hypothesis is proposed based on these findings:

H4: Participants' trust in the trainer will positively influence their continuation of the exercise.

### 2.3 Effect of training satisfaction on continuing exercise

Satisfaction is the sense of contentment or fulfilment without deficiency (National Institute of Korean Language, 2025). In the context of training, training satisfaction refers to participants' perception of being fully supported by the trainer, with no deficiencies. Ha and Kim (2021) define coaching satisfaction as the mutual trust between trainers and participants, reflecting participants' overall satisfaction with the trainer's behaviours and guidance throughout the learning process. Similarly, Mu and Jeong (2024) describe coaching satisfaction as the learner's emotional response towards the instructor, emphasising that instructors can improve satisfaction by demonstrating professional knowledge, technical competence, sincerity, and instructional methods tailored to participants' needs.

Continuing exercise refers to an individual's psychological commitment and consistent behaviour in engaging in physical activity. It involves not only the frequency, duration, and intensity of exercise (Dishman and Gettman, 1980) but also emotional attachment and persistence despite external challenges, such as time constraints or financial difficulties. In essence, it reflects the participant's commitment to integrating physical activity as a meaningful part of life.

Several studies have shown a significant relationship between training satisfaction and continued exercise. Mu and Jeong (2024) found that learners' satisfaction with the instructor positively influenced their intention to continue exercising. They recommended that instructors develop structured lesson plans and adopt engaging, learner-centred teaching methods to encourage sustained participation. Similarly, Cho and Kim (2017) discovered that satisfaction in physical education classes significantly improved adherence, describing it as a psychological outcome of instructor–learner interaction and a key factor reflecting the overall effectiveness of instruction.

**Figure 1:**
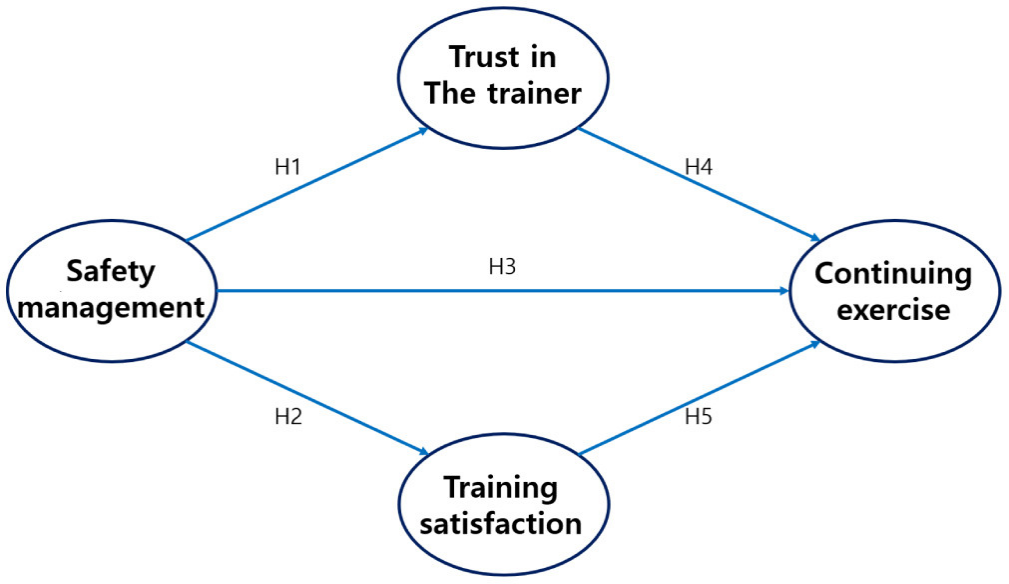
Proposed research model illustrating the relationships among safety management, trust in the coach, training satisfaction, and continuing exercise.

Based on these findings, the following hypothesis is proposed:

H5: Satisfaction with the golf trainer will positively influence participants' continuation of the exercise. The research model is presented in Figure 1.

## 3 Methods

### 3.1 Research participants

This study involved adults who participated in classes at an indoor driving range with a golf simulator in Seoul, Korea. Participants were recruited through convenience sampling. After obtaining written consent from 215 individuals, a survey was conducted, with 202 participants included in the study. Thirteen individuals were excluded due to unreliable responses, such as missing answers, repeated numbers, or insincere responses. The participants' characteristics are shown in Table 1. All participants provided informed consent prior to their inclusion in the study. The study adhered to the Declaration of Helsinki and was approved by the Korea National Institute for Bioethics Policy (approval no. P01-202411-01-009).

**Table 1:** Demographic characteristics of the study participants (*n* = 202).

**Characteristic**	**Categories**	**Frequency (*n*)**	**Percentage (%)**
Sex	Male	135	66.8
	Female	67	33.2
Age	20s	57	28.2
	30s	47	23.3
	40s	67	33.2
	50s	26	12.9
	Over 60	5	2.5
Handicap	28 or higher	21	10.4
	18 to 27	36	17.8
	8 to 17	33	16.3
	7 or less	112	55.4
Experience	<3 years	40	19.8
	<6 years	28	13.9
	<9 years	22	10.9
	<12 years	15	7.4
	12 years or more	97	48.0

### 3.2 Research instrument

A structured questionnaire was developed using validated instruments from prior research and adapted to fit the study context. It included 30 items: 4 on demographic characteristics, 8 on safety management, 7 on trust in the coach, 6 on training satisfaction, and 5 on continuing exercise (see Table 2). All items were rated on a 5-point Likert scale (1 = strongly disagree, 5 = strongly agree).

**Table 2:** Questionnaire content.

**Indicator**	**Content**	**Number of items**
General characteristics	Sex, age, handicap, and experience	4
Train safety management	Facilities and equipment for safety	8
	Training for safety	
Trust in the trainer	Single factor	7
Training satisfaction	Single factor	6
Continuing exercise	Single factor	5
Total		30

#### 3.2.1 Safety management

This construct was assessed using 8 items adapted and modified from Shin (2015) to align with the objectives of this study. It included two subfactors: (1) safety facilities and equipment and (2) safety training. An example item is: “*My trainer ensures that the equipment and facilities are safe before the lesson begins*.”

#### 3.2.2 Trust in the trainer

Seven items, adapted from Song (2011), were used to measure learners' perceived trust in their coach. This variable was treated as a single-factor construct. An example item is: “*I believe my trainer acts in my best interest*.”

#### 3.2.3 Training satisfaction

Six items from Jang (2019) were adapted to assess satisfaction with the training received. Training satisfaction was treated as a single-factor construct. An example item is: “*I am satisfied with the training methods used by my trainer*.”

#### 3.2.4 Continuing exercise

This construct was measured using five items adapted from Park et al. (2014) and treated as a single-factor construct to assess the intention to continue playing golf. An example item is: “*I plan to continue playing golf regularly*.”

### 3.3 Validity and reliability of the instrument

To ensure content validity, a panel of experts, Ph.D. holders with extensive quantitative research experience in sports science, reviewed the questionnaire items for clarity and relevance. Consistent with previous studies (Kim et al., 2009; Hair et al., 2010), acceptable fit is indicated by a TLI >0.90, SRMR below 0.07, and RMSEA <0.10. Confirmatory factor analysis (CFA) assessed construct validity, and the results indicated a satisfactory model fit [χ^2^(265) = 533.917, *p* < 0.001; SRMR = 0.046; TLI = 0.921, CFI = 0.930, RMSEA = 0.071].

Convergent validity was assessed using composite reliability (CR), average variance extracted (AVE), and Cronbach's alpha. Bae (2017) and Song (2015) suggest that convergent validity is confirmed when CR exceeds 0.70, AVE exceeds 0.50, and Cronbach's alpha exceeds 0.70. The results indicated CR values ranging from 0.841 to 0.935, AVE values from 0.571 to 0.711, and Cronbach's alpha values from 0.825 to 0.920, confirming the instrument's convergent validity and internal consistency. The results are shown in Table 3.

**Table 3:** Validity and reliability analysis.

**Variable**		**Items**	**λ**	**S.E**.	**CR(*t*)**	**CR**.	**AVE**	**α**
Safety management	Facilities and equipment for safety	I believe that the trainer maintains the equipment and instruments used in the classroom.	0.823			0.908	0.711	0.902
		I believe that the trainer allocates a dedicated space to store equipment and tools for proper use.	0.848	0.071	14.419			
		I believe that the trainer ensures there is sufficient space between equipment in the warehouse where items are stored.	0.859	0.082	14.692			
		I believe that the trainer is familiar with the safety rules for various facilities.	0.821	0.082	13.742			
	Training for safety	The train guides us to a location where we can receive medical attention or rest in case of a safety incident.	0.656			0.841	0.571	0.825
		The trainer incorporates warm-up exercises before every class.	0.739	0.128	9.138			
		The trainer issues appropriate warnings about dangerous behaviours during lessons.	0.832	0.110	10.047			
		The trainer adjusts the workout intensity based on the participants' physical conditions during the class.	0.764	0.106	9.399			
Trust in the trainer		I am confident that my trainer treats me fairly.	0.838			0.935	0.674	0.920
		I have confidence in my trainer's conscientiousness.	0.750	0.064	12.523			
		I have a strong sense of loyalty to my trainer.	0.740	0.065	12.285			
		I will support my trainer in any crisis.	0.855	0.063	15.353			
		I have confidence in my trainer's ability to teach golf.	0.788	0.059	13.488			
		I have open and honest communication with my coach.	0.777	0.067	13.199			
		My trainer would never attempt to deceive students for personal gain.	0.776	0.070	13.164			
Training satisfaction		I am content with my trainer's creative guidance.	0.819			0.930	0.689	0.913
		I am content with my trainer's accountability.	0.775	0.070	12.802			
		I am content with my trainer's teaching methods.	0.787	0.065	13.087			
		I am content with my trainer's instruction on golf rules and etiquette.	0.823	0.073	13.981			
		I am content with my trainer's golf course strategy.	0.811	0.066	13.666			
		I am content with my trainer's instruction on golf techniques.	0.773	0.067	12.760			
Continuing exercise		I feel confident during my exercise routine.	0.877			0.849	0.585	0.839
		I can manage challenging workouts well.	0.808	0.065	13.410			
		Exercise is my hobby.	0.699	0.074	10.983			
		It is convenient for me to exercise in terms of locations and facilities.	0.620	0.075	9.386			

X^2^ = 533.917; df = 265; *p* < 0.001; SRMR = 0.046; TLI = 0.921; CFI = 0.930; RMSEA = 0.071.

λ, Unstandardized Regression Weights; S.E., Standard Error; CR(t), Critical Ratio; *p, p*-value; AVE, Average Variance Extracted; CR, Construct Reliability; ɑ, Cronbach' ɑ.

### 3.4 Data processing

Data were analysed using IBM SPSS Statistics 21 and AMOS 21. Frequency analysis described the participants' demographic characteristics. An exploratory factor analysis (EFA) identified the underlying factor structure, which guided the subsequent CFA. CFA validated the measurement model and assessed model fit. CR and AVE evaluated convergent validity, while Cronbach's alpha coefficients assessed the internal consistency of each scale. Pearson correlation analysis examined the relationships among the variables. Finally, structural equation modelling (SEM) tested the hypothesised relationships among the constructs.

## 4 Results

### 4.1 Correlation analysis

Pearson's correlation analysis was performed to assess the relationship between each subfactor before hypothesis testing, with results shown in Table 4. A statistically significant correlation was found between facilities and equipment for safety, safety training, trust in the trainer, training satisfaction, and continuing exercise (*p* < 0.01). According to Kline (2011), a correlation coefficient above 0.85 suggests multicollinearity. In this study, the observed correlation ranged from 0.455 to 0.848, thus mitigating any concerns about multicollinearity.

**Table 4:** Correlation analysis results.

**Variable**		**Mean**	**Standard deviation**	**1**	**2**	**3**	**4**	**5**
Safety management	Facilities and equipment for safety (1)	4.153	0.863	1				
	Training for safety (2)	4.242	0.790	0.779^**^	1			
Trust in the trainer (3)		4.295	0.745	0.714^**^	0.769^**^	1		
Training satisfaction (4)		4.300	0.747	0.820^**^	0.792^**^	0.848^**^	1	
Continuing exercise (5)		4.134	0.795	0.455^**^	0.561^**^	0.673^**^	0.577^**^	1

^**^p < 0.01.

### 4.2 Verification of research model fit

SEM was used to assess the research model's fit. The results showed that the fit criteria were met: *x*^2^(df) = 318.922(147)/*p* < 0.001, SRMR = 0.042, TLI = 0.932 CFI = 0.941, RMSEA = 0.076, as presented in Table 5. Thus, the model was confirmed as appropriate. Additionally, the model explained 98.0% of the variance in training satisfaction, 84.7% in trust in the trainer, and 60.9% in continuing exercise.

**Table 5:** Model fit and hypothesis verification results.

**Hypothesis**	**Path**			**λ**	**S.E**.	**C.R**.	**Sig**.	**Result**
H1	Safety management	= >	Trust in the trainer	0.920	0.073	13.727	0.001	Accepted
H2	Safety management	= >	Training satisfaction	0.990	0.072	14.402	0.001	Accepted
H3	Safety management	= >	Continuing exercise	−0.832	1.905	−0.502	0.616	Rejected
H4	Trust in the trainer	= >	Continuing exercise	1.075	0.287	4.406	0.001	Accepted
H5	Training satisfaction	= >	Continuing exercise	0.501	1.711	0.323	0.747	Rejected
	R^2^: Trust in the trainer			0.980				
	R^2^: Training satisfaction			0.847				
	R^2^: Continuing exercise			0.609				
Model fit			*X*^2^ = 318.922, df = 147, *p* < 0.001, SRMR = 0.042, TLI = 0.932, CFI = 0.941, RMSEA = 0.076.					

λ, Unstandardized Regression Weights; S.E., Standard Error; CR(t), Critical Ratio.

### 4.3 Hypothesis verification

**Figure 2:**
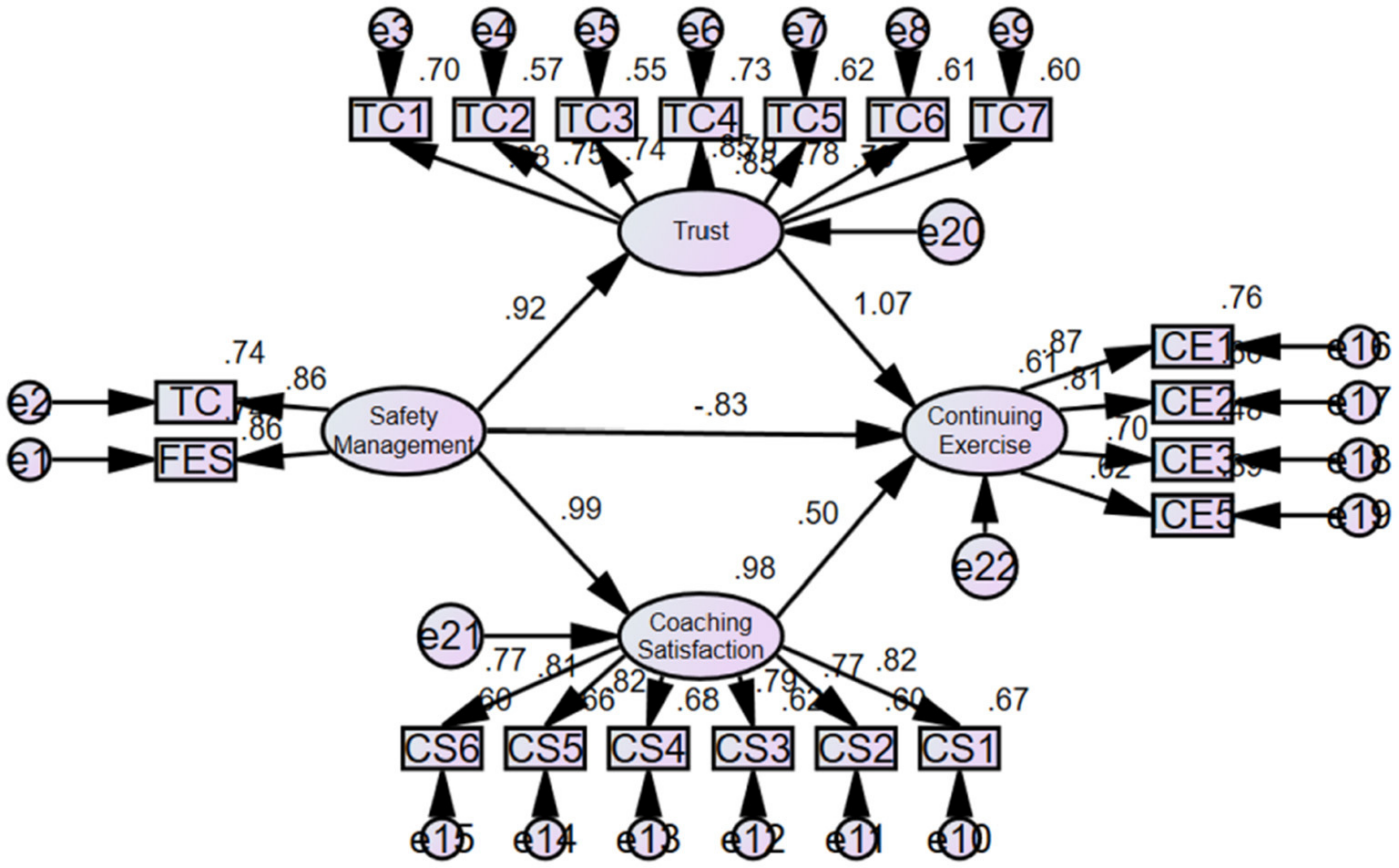
Results of the structural equation model, including standardised path coefficients and model fit indices.

The results of this study are as follows. The results are presented in Table 5 and Figure 2.

First, the causal relationship between safety management and trust in the trainer was analysed. Safety management had a statistically significant positive effect on trust in the trainer, with an unstandardised coefficient of 0.920, *t* =13.727 (*p* < 0.001). Therefore, the first hypothesis (H1) was accepted.

Second, the causal relationship between safety management and training satisfaction was analysed. Safety management had a statistically significant positive effect on training satisfaction, with an unstandardised coefficient of 0.990 (*t* = 14.402, *p* < 0.001). Therefore, the second hypothesis (H2) was accepted.

Third, the causal relationship between safety management and continued exercise was examined. The unstandardised coefficient of −0.832, *t* = −0.502, indicated no statistically significant effect on continuing exercise (*p* > 0.05). Therefore, the third hypothesis (H3) was rejected.

Fourth, the relationship between trust in the trainer and continued exercise was assessed. The unstandardised coefficient of 1.075, *t* = 4.406, showed a statistically significant positive effect of trust in the trainer on continued exercise (*p* < 0.001). Therefore, the fourth hypothesis (H4) was accepted.

Fifth, the causal relationship between training satisfaction and continuing exercise was analysed. The unstandardised coefficient of 0.501, with *t* = 0.323, revealed no statistically significant effect of training satisfaction on continuing exercise (*p* > 0.05). Therefore, the fifth hypothesis (H5) was rejected.

We conducted a bootstrapping analysis to assess the mediating effect of training satisfaction in the relationship between golf trainers' safety management and participants' continued exercise. Following Hayes (2013) and Bae (2017), we evaluated the mediation effect by comparing the chi-square (χ^2^) values and degrees of freedom of partial and full mediation models. A Δχ^2^ value of 3.84 or less with 1 degree of freedom indicates full mediation. We performed a bootstrapping procedure with 2,000 resamples and a 95% confidence interval. The results showed that training satisfaction fully mediated the relationship between safety management and continued exercise. Specifically, the comparison between the partial and full mediation models revealed a Δχ^2^ = 0.305 with Δdf = 1, and the indirect effect was statistically significant (*p* < 0.01). The results are shown in Table 6.

**Table 6:** Mediating effect analysis through bootstrapping.

**Path**				**Bootstrap estimate 95% confidence interval**				
				**Indirect effect (sig)**		**Lower (LLCI)**		**Upper (ULCI)**	
Safety management = > trust in the trainer = > continuing exercise				0.662 (0.001)		0.524		0.776	
**Model**		***X*** ^2^	**df**	***p***	**SRMR**	**TLI**	**CFI**	**RMSEA**
Complete mediation		319.227	148	0.001	0.042	0.932	0.942	0.076

Δχ^2^ = 0.305, Δdf = 1.

## 5 Discussion

The study aimed to examine the structural relationships among golf trainers' safety management, participants' trust in the trainer, training satisfaction, and continuing exercise. The findings are discussed below.

First, golf trainers' safety management was found to positively impact participants' trust in the trainer. Since the COVID-19 pandemic, golf participants in Korea have surged, along with a rapid expansion of golf-related businesses. While this growth promotes public health and lifelong physical activity, it has also resulted in frequent safety incidents at golf facilities. These incidents are partly attributed to inadequate safety awareness and insufficient education among golf participants (Jeong and Ryu, 2023). In this context, the study supports the view that proactive safety management, such as proper equipment handling, secure practise spaces, and addressing risky behaviours, enhances participants' trust in the trainer. Although research directly linking safety management and coaching trust is limited, the findings align with Jeong and Kim (2016), who argued that injury prevention and safe training environments strengthen instructor–learner relationships and trust. Similar conclusions were drawn in organisational safety research, where safety leadership positively influenced organisational trust (Choo et al., 2022; Lim and Lee, 2023). Furthermore, Jung and Kim (2020) highlighted safety management as a core competency for sports educators, particularly in unpredictable physical activity settings. These findings suggest that golf trainers should prioritise ongoing safety management training to build participants' trust.

Second, safety management also positively affected training satisfaction, consistent with previous studies (Lee et al., 2019; Lee and Kim, 2023). This includes safe facilities, equipment, and instruction, which enhance satisfaction across physical, psychological, and social domains. In this study, training satisfaction was defined as participants' evaluations of the trainer's responsibility, instructional methods, and technical guidance. Participants viewed the trainers' management of equipment, communication of safety rules, and adjustment of training intensity as for creating a safe and reliable learning environment. Considering that golf involves clubs and the risk of injury from swings, ball impact, or slips, safety is a key aspect of instructional quality. As human factors often contribute to safety incidents in sports (Lee and Park, 2006), safety education is particularly important for novice learners. These findings underscore the importance of effective safety management in enhancing training satisfaction, especially when trainers guide the safe use of equipment and foster a secure environment.

Third, contrary to expectations, golf trainers' safety management did not significantly affect participants' intention to continue exercising. This contrasts with previous studies that found a positive association between trainers' safety management and continued exercise (Ha and Park, 2023; Seo and Woo, 2025). This surprising result invites a deeper look into the sociocultural context of Korean adult learners. Although this result may seem counterintuitive, it can be understood within the context of Korea's unique golf instruction environment and the characteristics of the study's participants. Most learners in this study were adults who paid significant fees for short-term lessons from certified teaching professionals. While beginner golfers receive instruction on a range of skills, including safety, intermediate and advanced learners often seek specific technical guidance and may view safety management as irrelevant or restrictive. Consequently, safety management may be perceived as unnecessary interference, leading to a negative perception that affects motivation to continue.

It is important to note that the study's findings do not suggest that safety management should be excluded from instruction. Instead, the observed indirect effects are more significant: while safety management did not significantly affect the intention to continue exercise, it positively influenced both trust in the trainer and training satisfaction, which subsequently enhanced exercise adherence. This mediating pathway indicates that safety management indirectly supports sustained participation by fostering trust and satisfaction. Thus, instructors should consider safety management not just as a means to increase adherence but as a crucial component in safeguarding learners' health and wellbeing. Ultimately, as this study demonstrates, effective safety management enhances trust and satisfaction, fostering long-term commitment to golf instruction.

Fourth, this study found that participants' perceived trust in their golf trainer significantly influenced their intention to continue exercising. Trust played a crucial role in fostering sustained participation by creating a psychologically safe and reliable learning environment. Participants believed their trainer acted fairly, prioritised their wellbeing, and demonstrated professionalism—factors that kept them motivated despite the challenges of learning golf. This finding aligns with social exchange theory (Blau, 1964), which posits that relationships are built and sustained through reciprocal exchanges (Cropanzano et al., 2017). When participants feel supported and respected by their trainer, they are more likely to reciprocate through continued engagement, making trust essential for fostering loyalty and long-term commitment.

Prior research supports this interpretation. Kim and Jeong (2022) found that participants were more likely to maintain exercise routines when they trusted their coach to help them achieve their goals. Similarly, studies in various sports, such as golf, Pilates, and martial arts, have highlighted the role of trust in sustaining participation (Kim and Jeong, 2020; Kim, 2022; Kong, 2022). Berry (1995) and Kouzes and Posner (2017) noted that trust develops gradually through consistent and meaningful interactions. Therefore, golf trainers should cultivate trust not only through technical expertise but also by offering emotional support, fostering open communication, and addressing individual learner needs. These efforts can improve satisfaction and strengthen long-term participants' commitment.

Fifth, this study found that training satisfaction did not significantly affect the intention to continue exercise, which contrasts with previous research suggesting that training methods, performance outcomes, and learning environments positively influence satisfaction and engagement (Yoo and Hwang, 2015; Kim and Jeong, 2020; Hwang and Lee, 2021). A potential explanation for this discrepancy is the sociocultural context of adult golf participation in Korea, where many learners invest in high-cost private lessons focused on short-term skill acquisition (Kim and Han, 2023). Intermediate and advanced learners, in particular, often prioritise tangible performance improvements over emotional satisfaction or instructional style. Therefore, even if participants report high satisfaction with the training, this does not necessarily translate into a stronger intention to continue exercising.

Korean sports culture often prioritises practicality and the pursuit of personal goals, such as business networking or enhancing social status, over emotional connexion or community experience (Korea Consumer Agency, 2021). In this study, training satisfaction was assessed based on participants' perceptions of the instructor's responsibility, teaching methods, and skill delivery. However, when these factors are not perceived as contributing to the learner's individual goals, even high satisfaction may not result in sustained participation. This finding aligns with the Self-Determination Theory (Deci and Ryan, 2000), which argues that satisfaction is inadequate for behaviour maintenance. Long-term participation is more likely when core psychological needs—autonomy, competence, and relatedness—are fulfilled.

Finally, this study found that trust in the trainer fully mediates the relationship between safety management and intention to continue exercise. This indicates that safety management by golf instructors does not directly influence learners' intentions to continue exercising; instead, it indirectly promotes continued participation by enhancing trust in the trainer. In other words, learners do not develop the intention to continue exercising merely by perceiving that a safe environment has been established; active engagement in exercise requires formation of trust that is built through interactions with the instructor.

According to Ha and Park (2023), when learners perceive that the facilities they use as generally safe and recognise that instructors actively promote safety through clear guidelines and preventive actions during instruction, they develop greater safety satisfaction and trust in the instructor. As a result, they actively recommend the facilities and instructors to others. Therefore, this study highlights the need for thorough preparation in golf education settings, including the installation of protective equipment and clear signage to prevent accidents and safety education for learners led by instructors. Such efforts by golf facilities and instructors ultimately increase learners' trust in both the facility and the instructor, thereby strengthening their intention to continue exercising.

Consequently, changes in the current safety education programs for golf instructors in Korea is necessary. Specifically, safety education provided by organisations such as the Safety Management Corporation, which currently offer uniform, formal safety education programs targeting all sports, should be adapted to address the specific characteristics of golf. For example, preparing materials based on previous accidents at indoor and outdoor golf courses and installing safety signage and precautions at golf education sites will be useful. These changes will not only provide practical, field-centred safety education tailored to golf, but also reinforce trust in golf facilities and instructors and enhance learners' intentions to continue exercising. This further underscores the need to revise and adapt golf instructor education programs to provide on-site, tailored safety education.

In this context, Murray et al. (2018) emphasised the need for golf instructors to receive practical training that includes safe play, injury prevention, and inclusivity, presenting it as an essential condition for ensuring the health and safety of participants and fostering a sustainable environment for continued participation in the sport.

## 6 Conclusion

This study empirically examined the structural relationships among golf trainers' safety management, participants' trust in the trainer, training satisfaction, and exercise continuation intention. The results revealed that safety management positively influenced trust and training satisfaction, while trust significantly enhanced the intention to continue exercising. Although safety management did not significantly affect exercise continuation intention, its indirect effects through trust was positive. Specifically, trust in the trainer fully mediated the relationship between safety management and exercise continuation, suggesting that a safe environment fosters sustained participation when perceived as a positive and meaningful training experience. These findings emphasise the need for a holistic approach that encompasses not only physical safety but also emotional and educational support. The results also offer practical insights for developing instructional strategies within the golf education field.

## 7 Limitations and future research

Although this study provides valuable insights, several limitations must be acknowledged.

First, this study utilised convenience sampling targeting adult learners attending indoor golf training centres in Seoul. Therefore, generalisability of the findings to other regions or outdoor golf environments is limited. Future research should consider including a more diverse sample by taking into account factors such as age, location, and instructional environments (indoor vs. outdoor) to enhance external validity. In addition, conducting longitudinal studies that measure how trust and the intention to continue participation evolve over time to establish the causal relationships more clearly among these variables is necessary.

Second, safety management in this study was conceptualised in two dimensions: physical safety and instructional (training-centred) safety. However, other critical aspects, such as psychological safety, risk perception, and digital safety in simulator-based instruction, were not considered. Future research should address these evolving concepts, incorporating technological advancements and changes in instructional settings to develop a more comprehensive safety management framework.

Third, the discussion of this study was limited owing to a lack of previous research investigating the relationships among the variables. Therefore, future follow-up qualitative research, such as direct observations or in-depth interviews, to explore these variables more comprehensively will be valuable.

Fourth, the study relied on self-reported survey data, which may be influenced by social desirability bias and recall errors. To enhance the validity and reliability of future findings, researchers should incorporate objective indicators, such as actual exercise adherence or records of safety incidents, along with behavioural observations.

Fifth, it may also be meaningful to compare perceptions of safety across different countries to examine potential differences in cultural and economic contexts.

In conclusion, future research should adopt a wider range of methodological approaches to investigate the complex pathways through which safety management affects continued participation in sports.

## Data Availability

The raw data supporting the conclusions of this article will be made available by the authors, without undue reservation.
